# Patient Preferences and Values in Decision Making for Migraines: A Systematic Literature Review

**DOI:** 10.1155/2021/9919773

**Published:** 2021-09-17

**Authors:** Xianpeng Xu, Qingjie Ji, Min Shen

**Affiliations:** ^1^Department of Acupuncture and Moxibustion, Quzhou Hospital of Traditional Chinese Medicine, Quzhou, China; ^2^Department of Neurology, The First Affiliated Hospital of Zhejiang Chinese Medical University, Hangzhou, China

## Abstract

**Objective:**

To comprehensively summarize the evidence on the preferences and values of migraine patients.

**Methods:**

We searched PubMed, Embase, Web of Science, China National Knowledge Infrastructure, Sino-Med, Chongqing VIP, and Wanfang Data for studies on the preferences and values of migraine patients. A qualitative review was performed, but no quantitative synthesis.

**Results:**

Twenty‐one studies were finally included, involving a total of 8701 participants. Patients expected a cure, to be symptom-free, a reduction in frequency of headaches, a reduction in severity of headaches, and an improved quality of life from their preventive treatment. Patients expected rapid pain relief, complete pain relief, return to normal activities, no recurrence, and no adverse events from their acute symptomatic treatment.

**Conclusion:**

Efficacy is the primary consideration in the treatment of migraine. Specifically, the most important embodiment of patient preferences and values is the reduced frequency of attacks with preventive treatment as well as prompt analgesia with acute symptomatic treatment.

## 1. Introduction

Patient preferences and values are mostly convergent with those of healthcare workers, but there are also differences [1]. As direct recipients in the process of disease diagnosis and treatment, the preferences and values of patients cannot be ignored [2]. Patients themselves have expressed interest in the decision-making process, and their adherence to treatment can be simultaneously improved when they participate in the decision-making process [3, 4]. Evidence-based medicine states that optimal clinical decisions should take into account the experience of clinicians, clinical research evidence, and patient preferences and values [5]. Furthermore, evidence of patient preferences and values has also been emphasized in the development of guidelines [6–10].

Migraine is the third most prevalent disorder and the first cause of disability [11]. Current mainstay of migraine treatment is drugs, including prophylactic and analgesic drugs [12]. The use of prophylactic drugs aims to lessen the frequency and severity of the migraine attacks, and the common prophylactic drugs include antihypertensives (e.g., *β*-blockers, calcium channel blockers, and angiotensin-converting enzyme inhibitors), antidepressants, anticonvulsants, and antihistamines [12]. The use of analgesic drugs aims to prevent a migraine attack or to stop it once it starts, and the common analgesic drugs include triptans, nonsteroidal anti-inflammatory drugs, acetaminophen, combination (acetaminophen, caffeine, and aspirin), and narcotics [13]. Other treatments such as application of pressure, cold, or heat, acupuncture, and surgical treatment have also gradually attracted attention in recent years, but evidence support is still lacking [14].

According to the Grading of Recommendations, Assessment, Development, and Evaluation approach, patient preferences and values refer to the relative importance of the patient for the outcome or health state of interest [9]. Based on the experience of guideline experts [10], we defined patient preferences and values as the perspectives, expectations, and goals of patients regarding treatment attributes. Treatment attributes were divided into treatment process attributes and outcome attributes. Among them, treatment process attributes included treatment strategy, duration, route of administration, formulation, and cost; treatment outcome attributes included treatment benefits and side effects [10]. There had been a significant increase in the number of studies investigating the preferences and values in patients with migraines; to comprehensively summarize the evidence, we carried out this systematic literature study.

## 2. Methods

### 2.1. Eligibility Criteria

Inclusion criteria were as follows: studies related to patient preferences and values for migraine therapy, both the preventive treatment and the acute symptomatic treatment; studies that examined the context of the consideration of migraine therapy and how patients value alternative health states and experiences with treatment; and studies that examined the choices patients make when presented with decisional aids for management options regarding migraine therapy. The exclusion criteria were reviews, letters, posters, case reports, and case series.

### 2.2. Data Sources and Search Strategy

PubMed, Embase, Web of Science, China National Knowledge Infrastructure, Sino-Med, Chongqing VIP, and Wanfang Data were searched from their inception to August 2020. Search terms included migraine, patient preferences, patient values, and health attitude.  Table 1 provides a search strategy for the Embase database.

### 2.3. Study Selection and Data Extraction

Two investigators independently read titles, abstracts, and full text to identify eligible studies. Any conflicts were adjudicated through discussion. According to the characteristics of the included studies, we extracted the following basic information using a standardized data extraction form: the first author, year of publication, date of study conduction, type of study, number of patients, and their demographics (mean age, gender), treatment protocol, methods used for evaluating patient preferences and values, outcomes assessed, main results, and methodological characteristics.

### 2.4. Quality Assessment

An evaluation of the quality of the included studies was performed with the instrument recommended by the Agency for Healthcare Research and Quality (AHRQ) [15]. There were 11 items in the AHRQ checklist, and each item was evaluated using three evaluation options, yes (scored “1”), unclear (scored “0”), or no (scored “0”). The quality was classified into three levels: low quality = 0–3; moderate quality = 4–7; high quality = 8–11 [16].

### 2.5. Statistical Analysis

A qualitative review was performed, but no quantitative synthesis. The results are presented in tabular form.

## 3. Results

### 3.1. Results of Included Studies

**Figure 1:**
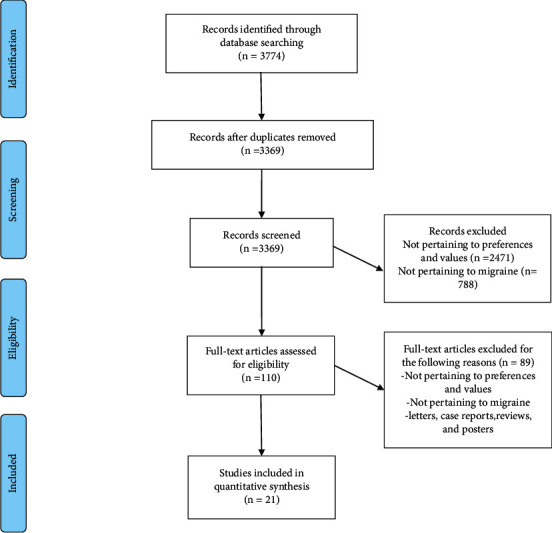
Flow diagram of the selection process.

A total of 3774 articles were acquired from the electronic search, and 405 duplicates were excluded. After screening the titles and abstracts, 3259 articles were excluded. Afterwards, the full texts of the remaining 110 articles were read for further evaluation, and 89 articles were excluded. Finally, a total of 21 studies [17–37] were ultimately included. The selection process is shown in Figure 1.

Characteristics of the studies, including date of study conduction, country, study design, simple size, migraine status, treatments, methods of stated-preference assessment, and methodological quality of the studies, are shown in Table 2. The most commonly used methods for evaluating patient preferences and values are described briefly in Table 3.

### 3.2. The Preventive Treatment for Migraine

Preferences and values for preventive treatment were reported in seven studies [17–23]. In general, all patients attached great importance to the preventive treatment of migraine. For treatment process, therapies with higher response rates, fewer adverse events, less frequent dosing regimens, and higher convenience were preferred [17, 18, 20, 22]. For treatment outcome, patients expected a cure, to be symptom-free, a reduction in frequency of headaches, a reduction in severity of headaches, and an improved quality of life from their treatment [18, 20, 21]. Efficacy was the most important aspect of outcome in preventive treatment; some patients even did not mind taking more than one preventive agent at one time if greater efficacy could be achieved [22]. The preventive treatment for migraine was important; however, not all patients actually used this treatment [19]. More details are shown in Table 4.

### 3.3. Acute Symptomatic Treatment for Migraine

Preferences and values for acute symptomatic treatment were reported in 14 studies [24–37]. For treatment process, therapies with a faster onset of action, a longer duration of the effects, fewer adverse events, and lower price were preferred [26–35, 37]. Triptans were the most commonly used drugs, and the order of priority for dosage form of triptans was tablets, nasal spray, and subcutaneous injection [24, 27, 30]. For treatment outcome, patients expected rapid pain relief, complete pain relief, return to normal activities, no recurrence, and no adverse events from their treatment [24, 27–37]. More details are shown in Table 5.

## 4. Discussion

A literature search yielded several published studies on preferences and values among patients with migraines. In this research, we systematically evaluated studies reporting the preferences and values of patients with migraines, thus providing summarized evidence for clinicians.

### 4.1. Summary of Main Findings

In this review, 21 studies enrolled 8701 participants were final included. In summary, evidence from these included studies suggested that the efficacy was the primary consideration in the treatment of migraine. For preventive treatment, therapies with higher response rates, fewer adverse events, less frequent dosing regimens, and higher convenience were preferred. Patients expected a cure, to be symptom-free, a reduction in frequency of headaches, a reduction in severity of headaches, and an improved quality of life from their preventive treatment. For acute symptomatic treatment, therapies with a faster onset of action, a longer duration of the effects, fewer adverse events, and lower price were preferred. Patients expected rapid pain relief, complete pain relief, return to normal activities, no recurrence, and no adverse events from their acute symptomatic treatment. Moreover, triptans were the most commonly used drugs for acute symptomatic treatment, and the order of priority for dosage form of triptans was tablets, nasal spray, and subcutaneous injection.

### 4.2. Regimens for Migraines

Preventive treatment for migraines should be preemptive, short term, or maintained. Antiepileptic drugs, *β*-blockers, antidepressants, calcium channel antagonists, botulinum neurotoxins, and serotonin antagonists are the most commonly used drugs for migraine prevention [12]. On the basis of evidence-based medical evidence, the first-line medications identified as effective include topiramate, divalproex, propranolol, metoprolol, and timolol; the second-line medications identified as effective include venlafaxine, amitriptyline, nadolol, and atenolol [38]. For migraine prevention, *β*-blockers are the most widely used drugs, which can reduce the frequency of attacks by more than 50%, and there are no absolute or relative contraindications [39]. Tricyclic antidepressants are also used to prevent migraines; however, only amitriptyline has proven efficacy in migraine. In addition, the high incidence of adverse events limits its use [40]. Since the efficacy of placebo-controlled trials has been confirmed, antiepileptic drugs are increasingly recommended for migraine. However, it is worth noting that most antiepileptic drugs may substantially interfere with the efficacy of oral contraceptives [41]. Furthermore, other medications such as calcium channel blockers, angiotensin-converting enzyme inhibitors, angiotensin receptor blockers, onabotulinumtoxinA, and complementary and alternative medicines cannot be recommended for migraine prevention due to the limited evidence quality [38].

For acute symptomatic treatment, the first-line medications for mild to moderate migraine are acetaminophen and nonsteroidal anti-inflammatory drugs, whereas triptans for moderate to severe migraines; for those with refractory migraine, dihydroergotamine and antiemetics are recommended for use as second- or third-line medications [42]. The use of acetaminophen and nonsteroidal anti-inflammatory drugs for mild to moderate migraine attacks is supported by strong evidence [42]. Moreover, nonsteroidal anti-inflammatory drugs have better efficacy than acetaminophen, but can cause gastric irritation or antiplatelet effects [43]. Triptans share a common mechanism of action and have strong evidence of effectiveness for moderate to severe migraine attacks [43]. However, different types of triptans have different routes of administration and kinetics, and they may be expensive [44]. Hence, appropriate individualized use is essential. Furthermore, other medications such as dihydroergotamine, opioids, and antiemetics have good evidence of effectiveness for migraine. However, they are reserved as second-line drugs due to adverse effects, abuse potential, route of administration, or cost [42].

### 4.3. Limitations

To the best of our knowledge, this is the first study to summary the evidence on the preferences and values of migraine patients. However, limitations should be acknowledged. First, the definition and eligibility criteria for preferences and values are broad; the lack of standardized methods for reporting and identifying the evidence of patient preferences places additional limitations on our research. Second, it is tentative and empirical to use a systematic literature review method to summary evidence; there might be nonrigorous and inconsistent phenomena.

## 5. Conclusions

In summary, evidence from these included studies suggests that the efficacy is the primary consideration in the treatment of migraine. Specifically, the most important embodiment of patient preferences and values is the reduced frequency of attacks with preventive treatment as well as prompt analgesia with acute symptomatic treatment.

## Figures and Tables

**Table 1:** Search strategy for Embase.

Query	Search term
#1	“Migraine”/exp
#2	“Migraine”:ab, ti OR “migraines”:ab, ti OR “status migrainosus”:ab, ti OR “sick headache”:ab, ti OR “sick headaches”: ab, ti
#3	#1 OR #2
#4	“Patient preference”/exp
#5	“Patient^*∗*^ preference^*∗*^”:ab, ti OR “patient^*∗*^ expectations^*∗*^”:ab, ti OR “patient^*∗*^ perspective^*∗*^”: ab, ti OR “patient^*∗*^ perception^*∗*^”:ab, ti OR “patient^*∗*^ decision^*∗*^”: ab, ti OR “patient^*∗*^ value^*∗*^”: ab, ti OR “patient^*∗*^ view^*∗*^”:ab, ti OR “user^*∗*^ view^*∗*^”: ab, ti
#6	“Attitude to health”/exp
#7	“Attitude to health“: ab, ti OR “health attitude^*∗*^”: ab, ti
#8	#4 OR #5 OR #6 OR #7
#9	#3 AND #8

**Table 2:** The preventive treatment of migraine.

Study	Date	Country	Study design	Simple size	Migraine status	Treatment					Method	Score
						Drug	Usage	Duration	Cost	Side effect		
Cowan et al. [17]	2016–2017	American	Cross-sectional	417	≥1 d/m	A new class of biologics	Subcutaneous injection	Administer monthly or quarterly	Disregarded	Unclear	Ranking	9
Mansfield et al. [18]	February and May, 2017	American	Cross-sectional	100	≥6 d/m		Injection or oral pill	Once/m, once/d, twice/m	$5–$175/m	Yes	SG	10
Kol et al. [19]	2008^a^	Netherlands	Cross-sectional	151		Triptans, analgesics				Yes	SG	9
Peres et al. [20]	2007^a^	American	Cross-sectional	250	≥12 m/y	*β*-blockers, calcium channel blockers, antidepressants, antiepileptics, neurotoxins		Once/3 m, once/d, twice/d	Out-of-pocket expense	Yes	Ranking	8
Kelman [21]	2006^a^	American	Prospective study	1750						Yes	Interviews	8
Rozen [22]	2006^a^	American	Cross-sectional	150		Analgesic, natural therapy					Ranking	9
Wenzel et al. [23]	September to November, 2002	American	Cross-sectional	22		Over the counter		Once/d	Low price		Ranking	9
Lipton and Stewart [24]	1998	American	Cross-sectional	688			Capsule, subcutaneous injection			Yes	Interviews	7
Adelman et al. [25]	January to June, 1999	American	Prospective study	367		Rizatriptan	Disintegrating tablet, conventional tablet.	10 mg/d, 6 m			Interviews	8
Pascual et al. [26]	2001^a^	Spain	Case-control study	94		Sumatriptan, zolmitriptan	Oral tablet	50 mg, 2.5 mg			Ranking	8
Weidmann et al. [27]	2003^a^	American	Case-control study	33	2–6 d/m	Sumatriptan	Oral, intranasal, subcutaneous		$45	Yes	Ranking	9
Dahlöf et al. [28]	2002–2003	Sweden	Case-control study	232	Acute attacked	Zolmitriptan	Oral, intranasal, subcutaneous	5 mg, 6 consecutive		Yes	TTO	9
Lipton et al. [29]	2005^a^	American	Cross-sectional	415		Triptan	Oral			Yes	TTO	8
Schoenen et al. [30]	2005^a^	American	Case-control study	323	Acute attacked	Eletriptan or sumatriptan	Oral, subcutaneous	80 mg, 6 mg		Yes	TTO	8
Lainez et al. [31]	2001–2002	Italy	Case-control study	372	Acute attacked	Rizatriptan, eletriptan		10 mg/40 mg			TTO	9
Dowson et al. [32]	2007^a^	United Kingdom	Prospective study	48	1–4 d/m	Zolmitriptan	Oral	2.5 mg, 5 mg; 10 m			TTO	8
Diez et al. [33]	2007^a^	Italy	Prospective study	436	2–6 d/m	Rizatriptan, almotriptan		12.5 mg, 10 mg		Yes	TTO	9
Lanteri et al. [34]	2003	France	Prospective study	1710	6 d/1.5 m	Triptans, analgesics, ergot derivatives					Ranking	9
Bartolini et al. [35]	2011^a^	Italy	Randomized controlled	133	1–6 d/m	Frovatriptan, almotriptan		2.5 mg, 12.5 mg			Ranking	10
Gonzalez et al. [36]	2013^a^	American	Cross-sectional	510							SG	8
Smelt et al. [37]	2014^a^	Netherlands	Cross-sectional	300		Triptans, ergotamine, analgesics					Ranking	8

TTO, time trade-off; SG, standard gamble. ^a^The date are year of publication, because survey dates were not reported.

**Table 3:** Description of the main methods used for evaluating patient preferences and values.

Name	Description	Example
Ranking	Researchers ask patients to rate a set of outcomes on an ordered ‘‘Likert-type” scale (rating) or to rank them from the most to the least important. Rating can also use visual analog scale and in this case utilities can be derived	From Ref. [17]: ‘‘patients were ranked on a seven-point scale, with 1 being “not at all likely” and 7 being “extremely likely,” their likelihood of acceptance of and adherence to the new medication in scenarios in which either monthly or quarterly dosing is available”
Time trade-off	Researchers ask patients to choose between the health states as described in a clinical scenario during *X* years and a shorter life in normal health. The duration *X* is varied until the patient is unable to choose between the two options	From Ref. [30]: ‘‘three attacks were treated on each study medication. Assessment of subjective preference was evaluated, after which patients freely choose which study medication they wished to use to treat each of the three additional migraine attacks”
Standard gamble	Researchers ask patients to choose between two possible outcomes: a suboptimal health state that is certain and a gamble with one better (for example, full health) and one worse (for example, death or side effects) outcome possible. The probability of the gamble is varied during the experiment and the point of indifference is used to derive the utility of the health state	From Ref. [18]: ‘‘respondents valued a change from a 10% reduction in headache days per month to a 50% reduction more highly than avoiding the worst levels of adverse events. Nevertheless, respondents were willing to forgo some improvements in efficacy for less-severe adverse events”

**Table 4:** Preferences and values for preventive treatment.

Study	Treatment process	Treatment outcome
Cowan et al. [17]	Most patients preferred monthly or quarterly dosing, while a small proportion had no preference	
Mansfield et al. [18]	Patients tended to inject monthly or daily rather than twice a month when treating	It was more important to change the number of migraine attack days from a 10% reduction to a 50% reduction than to avoid adverse events
Kol et al. [19]	Fifty-five percent of patients wanted to use prophylaxis; only 8% actually used this treatment	
Peres et al. [20]	Therapies with higher response rates, fewer adverse events, and less frequent dosing regimens were preferred	Patients rated efficacy as the most important aspect of preventive treatment outcome
Kelman [21]		A percentage of 95.2 expected a reduction in frequency of headaches from their treatment, 95.6% a reduction in severity of pain, 79.7% to be symptom-free, 27.8% a cure, and 95.5% an improved quality of life
Rozen [22]	If greater efficacy could be achieved, patients did not mind using more than 1 prophylactic agent	
Wenzel et al. [23]		The vast majority of patients wanted to use over-the-counter drugs to effectively prevent migraine

**Table 5:** Preferences and values for acute symptomatic treatment.

Study	Treatment process	Treatment outcome
Lipton and Stewart [24]	In terms of dosage form, the order of priority was tablets, nasal spray, and subcutaneous injection	Eighty-seven percent of patients had expected complete pain relief after treatment, 86% had no recurrence, and 83% had rapid pain relief
Adelman et al. [25]	Among the included patients, 188 chose orally disintegrating tablets, while 179 preferred conventional tablets	
Pascual et al. [26]	The reasons for patient preference for either of the two triptans were faster onset, duration of action, fewer adverse events, and lower price	
Weidmann et al. [27]	In terms of dosage form, the order of priority was tablets, nasal spray, and subcutaneous injection	Rapid pain relief without adverse events
Dahlöf et al. [28]	Most patients would like to continue using zolmitriptan nasal spray	Rapid pain relief without adverse events
Lipton et al. [29]	When selecting a triptan, the rating of efficacy attributes was clearly more important than tolerability or consistency	Absence of pain within 1 hour was the most desired treatment outcome for migraine patients
Schoenen et al. [30]	More patients preferred eletriptan	Rapid pain relief without recurrence
Lainez et al. [31]	More patients preferred rizatriptan 10 mg wafer compared to eletriptan 40 mg tablets	Rapid pain relief and return to normal activities
Dowson et al. [32]	At baseline, most patients indicated a preference for conventional tablets. After trying other formulations with a faster onset of action, most patients no longer preferred conventional tablets	Rapid pain relief
Diez et al. [33]	More patients preferred almotriptan 12.5 mg compared to rizatriptan 10 mg	Rapid pain relief and return to normal activities
Lanteri et al. [34]	Patients tended to use triptans and were satisfied with this treatment	Freedom from pain and rapid onset of action
Bartolini et al. [35]	Most of the patients indicated a preference for triptans	Rapid pain relief without recurrence
Gonzalez et al. [36].		No recurrence of headache
Smelt et al. [37]		Patients wanted to relieve pain within 30 minutes after treatment, returned to normal activity within 1 hour, and had no recurrence of headache

## Data Availability

All data generated or analyzed during this study are included in this published article.

## Abbreviations

ODT: Orally disintegrating tablet
TTO: Time trade-off
SG: Standard gamble.
